# Dietary intervention rescues maternal obesity induced behavior deficits and neuroinflammation in offspring

**DOI:** 10.1186/s12974-014-0156-9

**Published:** 2014-09-12

**Authors:** Silvia S Kang, Aishe Kurti, Damien A Fair, John D Fryer

**Affiliations:** Department of Neuroscience, Mayo Clinic Jacksonville, 4500 San Pablo Road, Jacksonville, FL 32224 USA; Neurobiology of Disease Program, Mayo Graduate School, 200 First Street SW, Rochester, MN 55905 USA; Department of Behavioral Neuroscience and Psychiatry, Oregon Health and Science University, Portland, OR 97239 USA; Advanced Imaging Research Center, Oregon Health and Science University, Portland, OR 97239 USA

**Keywords:** Maternal obesity, Dietary intervention, Neuroinflammation, Behavior, ASD, ADHD

## Abstract

Obesity induces a low-grade inflammatory state and has been associated with behavioral and cognitive alterations. Importantly, maternal environmental insults can adversely impact subsequent offspring behavior and have been linked with neurodevelopmental disorders such as autism spectrum disorder (ASD) and attention deficit hyperactivity disorder (AHDH). It is unknown if maternal obesity significantly alters offspring sociability, a key ASD feature, and if altering maternal diet will provide an efficacious intervention paradigm for behavioral deficits. Here we investigated the impact of maternal high fat diet (HFD) and maternal dietary intervention during lactation on offspring behavior and brain inflammation in mice. We found that maternal HFD increased anxiety and decreased sociability in female offspring. Additionally, female offspring from HFD-fed dams also exhibited increased brain IL-1β and TNFα and microglial activation. Importantly, maternal dietary intervention during lactation was sufficient to alleviate social deficits and brain inflammation. Maternal obesity during gestation alone was sufficient to increase hyperactivity in male offspring, a phenotype that was not ameliorated by dietary intervention. These data suggest that maternal HFD acts as a prenatal/perinatal insult that significantly impacts offspring behavior and inflammation and that dietary intervention during lactation may be an easily translatable, efficacious intervention to offset some of these manifestations.

**Electronic supplementary material:**

The online version of this article (doi:10.1186/s12974-014-0156-9) contains supplementary material, which is available to authorized users.

## Background

Maternal obesity is a pervasive health issue, with over 30% of child-bearing age women in the United States being categorized as obese [1]. Adverse symptoms such as gestational diabetes, pre-term birth, and birth injury are associated with maternal obesity [2-4]. Emerging data have also linked maternal obesity with long-lasting ramifications in offspring including altered immunity, metabolism, increased obesity, and cardiovascular risk [5-11]. Maternal obesity-induced changes in offspring brain lipid peroxidation, hippocampal neurogenesis, and inflammation [12-14] may also impact cognition and behavior.

Obesity is associated with increased risk of behavioral disorders. In humans, increased body mass index (BMI) has been connected with elevated anxiety and depression [15-18]. Interestingly, maternal obesity also increased anxiety in non-human primate and rodent offspring [14,19-21]. Altered maternal environments are implicated in neurodevelopmental disorders such as attention deficit hyperactivity disorder (ADHD) and autism spectrum disorder (ASD). ADHD symptoms in children have been associated with pre-pregnancy adiposity [22-24] and animal models of maternal high fat diet (HFD) revealed increased offspring activity [25]. Furthermore, children from mothers with a pre-pregnancy body mass index ≥ 35.0 exhibited delayed mental development as well as learning and behavioral disabilities [26,27]. In humans, maternal obesity or infection has also been linked to increased autism risk in children [28-30]. While rodent modeling of maternal infection results in offspring displaying features of ASD [31,32], it is unknown whether maternal obesity alone induces any ASD-like behaviors in subsequent offspring.

Growing evidence suggests a link between altered immunity and aberrant behavior and cognition [31,33,34]. Pro-inflammatory cytokines, including interleukin (IL)-1β and tumor necrosis factor (TNF)α, have been associated with increased anxiety, altered cognitive function [35-38], and are elevated in ASD patients [39-41]. Activation of microglia, the central nervous system (CNS) resident immune cells, often coincides with behavioral manifestations [14,42-45]. Although maternal obesity has been suggested to increase brain inflammation [14], intervention strategies that may alleviate maladaptive behaviors and CNS inflammation observed in offspring have not been explored.

Human data suggest that maternal obesity may be linked to ASD-like behaviors [30]; however, to date, little is known whether rodent models of maternal obesity will recapitulate any aspect of ASD in offspring and provide a model to examine the efficacy of intervention strategies. Additionally, it is unclear whether maternal intervention strategies would prove to be efficacious against neuroinflammation that is observed in offspring as a result of maternal HFD [14] in addition to any behavioral abnormalities. Here we investigated the effects of maternal obesity and maternal dietary intervention during lactation on offspring in a rodent model. Specifically, offspring behavior from HFD-fed dams without dietary intervention was assessed for behavioral abnormalities including anxiety, hyperactivity and sociability, as well as neuroinflammation. These data indicate that maternal obesity impacts several aspects of behavior, including sociability, and evokes neuroinflammation in offspring. Importantly, modulation of offspring behavior and neuroinflammation by maternal dietary intervention suggests that a significant alleviation of offspring abnormalities can be achieved using an easily translatable intervention strategy.

## Materials and methods

### Animals

C57BL/6 J mice (The Jackson Laboratory, Bar Harbor, ME, USA) were housed under standard laboratory conditions in ventilated cages on 12-hour light:dark cycles in a specific pathogen-free environment. Animal protocols were reviewed and approved by Mayo Clinic Institutional Animal Care and Use Committee.

### Diets and litters

Six-week-old female breeders were placed on either control diet (CD) (10% of calories from fat; Research Diets, New Brunswick, NJ, USA #D12450B) or HFD (60% of calories from fat; Research Diets New Brunswick, NJ, USA #D12492). Comparison of the diets is shown in Additional file 1: Table S1. Mice were fed *ad libitum* for six weeks prior to breeding with a two- to three-month-old male. Females were either maintained on the gestation diet or on post-natal day 0 were switched to the opposing diet for the duration of lactation. This created four diet conditions: CD/CD, HFD/HFD, HFD/CD and CD/HFD, indicating gestation/lactation diets respectively. To reduce the impact of litter effects, litters were adjusted to no more than nine per dam. The average litter sizes for the CD/CD = 5.2, HFD/HFD = 4.2, CD/HFD = 8.3 and HFD/CD = 4. The only significant difference was between CD/HFD versus HFD/HFD (*P* = 0.02) and HFD/CD (*P* = 0.03) using a one-way analysis of variance (ANOVA) with Tukey *post-hoc* testing. Litter size did not appear to impact subsequent pup weight or behavior. For example, linear regression analysis of litter size versus interaction score, an important parameter in the three-chamber social interaction assay, revealed no significant correlation between litter size and behavior (linear regression R^2^ = 0.02557, *P* = 0.177). Additionally, no associations were observed between litter size and pup weight (Additional file 2: Figure S1). The total number of dams used was CD/CD = 11, HFD/HFD = 8, CD/HFD = 4, HFD/CD = 5. For behavior, the number of male and female offspring used is presented in the graph. A subset was randomly selected for biochemistry. All offspring were weaned at postnatal day (P)21 and were placed on irradiated standard chow (Harlan Laboratories; Madison, WI, USA).

### Behavioral testing

Behavior was assessed between 9:00 am and 5:00 pm. For all behavioral tests, mice were acclimated in the room for an hour prior to the onset of testing. The apparatus was thoroughly cleaned with 30% ethanol between test mice to remove any residual odors in the equipment. For all behavioral tests, data were recorded and monitored using Fujinon cameras mounted overhead in conjunction with Anymaze tracking software (Stoelting Co.; Wood Dale, IL, USA). Specific regions of interest were defined digitally using Anymaze software for each test and the center body of the animals was subsequently tracked for the duration of the test in order to calculate behavioral measurements from the recorded visual files. Raw data produced by Anymaze software were then analyzed as described in section data analysis and statistics.

### Open field assay

Mice were placed in a 40 × 40 cm black Plexiglas box with a brightly lit center. Behavioral activity was recorded by Fujinon cameras for 15 minutes and was tracked using Anymaze software (Stoelting Co.; Wood Dale, IL, USA). An imaginary 13 cm × 13 cm region in the center of the box and a perimeter region were defined using Anymaze software. Center:total distance ratios were computed by Anymaze software by taking the total distance traveled in the pre-defined center region relative to the total distance traveled during the 15 minute observation period. Side mounted photobeams located 7.6 cm from the floor of the box were used to monitor rearing whereas other measures (for example. total distance traveled and center:total distance) were acquired using the overhead camera.

### Three-chamber social interaction test

Social behavior was tested in a 3-chamber Plexiglas 40 × 40 cm box consisting of two 17 × 40 cm regions separated by 2 dividers forming a smaller 5 × 40 cm center region. Mice were able to move freely through a small 8 × 5 cm opening that was aligned in both dividers. Each of the larger chambers contained an inverted wire mesh cylinder in opposing corners. Mice were initially acclimated to the box and empty cylinders for four minutes and then placed in temporary holding cages. A sex-matched probe mouse of the same strain was placed in one of the inverted mesh cylinders and the test mouse was placed back in the box. The open mesh enabled visual, olfactory, and auditory interactions between probe and test mice. Test mice were subsequently monitored for ten minutes in the presence of the probe mouse using Fujinon cameras and Anymaze software (Stoelting Co.; Wood Dale, IL, USA). Imaginary circular regions encompassing the empty cylinder and the probe mouse cylinder were defined using Anymaze software. Time in mouse cup minus time in empty cup region was then calculated using these pre-defined regions. Interaction scores, which normalize for the total time spent in both regions, were calculated by the following formula:

$$ \left(\mathrm{Tim}{\mathrm{e}}_{\mathrm{Mouse}\kern0.24em \mathrm{cup}}-\mathrm{Tim}{\mathrm{e}}_{\mathrm{e}\mathrm{mpty}\;\mathrm{cup}}\right)/\left( Tim{e}_{Mouse\kern0.24em  cup}+ Tim{e}_{empty\; cup}\;\right) $$

### Weight and general procedures

Female mice were weighed prior to the onset of diet initiation and then on a weekly basis prior to and through gestation. Offspring were weighed at weaning (P21) and prior to commencement of behavioral testing. For biochemistry and immunohistology, a subset of animals was harvested 24 hours after the final behavior test.

### Tissue processing

Animals were deeply anesthetized with pentobarbital prior to cardiac perfusion with PBS to expunge blood from the cerebrovasculature. For biochemical analysis, hemi-brain tissues were quickly frozen on dry ice until further processing. Tissues were briefly sonicated in Tris buffered saline with EDTA (TBSE) (50 mM Tris pH = 7.5, 150 mM NaCl, 1 mM EDTA) with 1X protease and phosphatase inhibitors (Thermo Scientific, Waltham, MA, USA). An aliquot of this sonicated tissue suspension was immediately placed into Trizol LS for RNA isolation using the Direct-zol RNA kit (Zymo Research, Irvine, CA, USA). Another aliquot was centrifuged for 15 minutes at 20,000 × *g* at 4°C and the soluble TBSE fraction was isolated for cytokine assessment. TBSE tissue protein levels were assessed using a BCA kit (Thermo Scientific, Waltham, MA, USA).

### Immunohistochemistry

Brains obtained from animals perfused with PBS followed by 10% normal buffered formalin (NBF) were further drop-fixed overnight in 10% NBF at 4°C. Samples were then switched to 30% sucrose in PBS and incubated overnight at 4°C. Fifty micron sagittal brain sections were cut on a freezing-sliding microtome and stored in cryoprotectant at −20°C until staining. Tissues were placed in netwells in a 12-well plate and washed with PBS to remove cryoprotectant. Sections were blocked for endogenous peroxidase activity and permeabilized with 0.6% H_2_0_2_, 0.1% NaN_3_ in PBS-X (1X PBS containing 0.3% Triton-X) for 30 minutes at room temperature (RT). Samples were washed x3 with PBS-X for 10 minutes/wash prior to blocking with 1% milk PBS-X for 90 minutes at RT. Sections were incubated with 1:5,000 Iba1 (catalog # 019-9741, Wako, Richmond, VA, USA) in 0.5% milk PBS-X for 2 days rocking at 4°C. After ×4 washes with PBS-X at RT, sections were incubated with the Vectastain kit anti-Rabbit IgG component (Vector Labs, Burlingame, CA, USA) for 2 days, rocking at 4°C. Samples were washed ×4 with PBS-X at RT and incubated with the ABC component for three hours. Iba1 staining was then developed using the DAB kit (Vector Labs, Burlingame, CA, USA) according to manufacturer’s instructions. Images were acquired using an Aperio XT Scanner (Aperio, Vista, CA, USA) at a ×20 magnification. Densitometric analysis of Iba1 staining intensity in the amygdala was performed with ImageJ software.

### ELISA

Tissue cytokine levels were measured from hemi-brain tissue using IL-1β and TNFα enzymelinked immunosorbent assay (ELISA) kits according to manufacturer’s instructions (Biolegend, San Diego, CA, USA), with values expressed as pg/mg of total protein.

### RT-qPCR

Total RNA was isolated from sonicated tissues using a Direct-zol total RNA isolation kit (Zymo Research, Irvine, CA, USA) according to manufacturer’s instructions with in-column DNase I treatment. RNA was reverse transcribed in first strand buffer containing 0.01 M dithiothreitol (DTT), 200 ng random hexamers, 0.5 mM dNTPs and 40U M-MLV according to M-MLV RT protocols (Invitrogen-Life Technologies, Grand Island, NY, USA). To conduct real time quantitative polymerase chain reaction (RT-qPCR), cDNA was added to a reaction mix (10 μL final volume) containing 300 nM gene-specific primers and Universal SYBR green supermix (Bio-Rad, Hercules, CA, USA). All samples were run in triplicate and were analyzed on an ABI 7900 HT Fast Real Time PCR instrument (Applied Biosystems-Life Technologies, Carlsbad, CA, USA) for quantitative monitoring of PCR product formation. Relative gene expression was normalized to *Gapdh* controls and assessed using the 2^-ΔΔCT^ method. Primer sequences are as follows: *Gapdh*: F: CTGCACCACCAACTGCTTAG; *Gapdh*: R: ACAGTCTTCTGGGTGGCA GT: Iba1: F: GGATTTGCAGGGAGGAAAAG; Iba1: R: TGGGATCATCGAGGAATTG.

### Data analysis and statistics

Data were analyzed using Prism statistical software (La Jolla, CA, USA). For maternal weight gain, data are analyzed as a repeated measures ANOVA. Behavioral data were assessed within each sex by one-way ANOVA tests using a Fisher’s LSD *post-hoc* analysis. Biochemical data were examined using one-way ANOVA with Tukey *post-hoc* analysis. All data with *P* < 0.05 were considered significant.

## Results

### Maternal high fat diet increases weight in both dams and offspring

**Figure 1:**
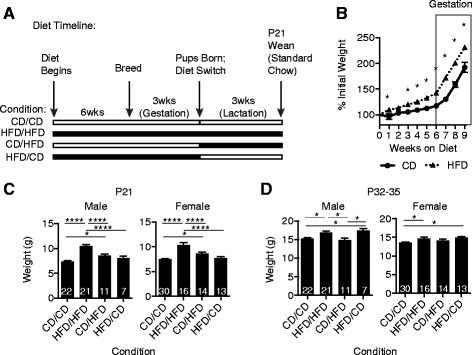
**High fat diet (HFD) significantly increases maternal and offspring weight. (A)**, Timeline for feeding schedule of HFD (60% calories from fat; black line) or control diet (CD) (10% calories from fat; white line) for the experimental conditions. **(B)** Percent initial weight for dams fed CD (black line, circle) or HFD (black dotted line, triangle) 6 weeks prior to and during gestation represented as mean ± SEM. N = 12 to 14 per group, *P < 0.01 (weeks 1 to 3), *P < 0.001 (week 4) or *P < 0.0001 (weeks 5 to 9), repeated measures ANOVA. **(C-D)** Mean weight ± SEM for male and female offspring from CD/CD, HFD/HFD, CD/HFD or HFD/CD dams at (C), P21 and (D), P32-35 (N = 7 to 22 or N = 13 to 30 per condition for males and females, respectively; one-way ANOVA with *post-hoc* Tukey analysis; P21 males *P = 0.0156, ****P < 0.0001; P21 females *P = 0.045, ****P < 0.0001; P32-35 males CD/CD versus HFD/HFD *P = 0.0169, CD/CD versus HFD/CD *P = 0.0319, HFD/HFD versus CD/HFD *P = 0.0113, CD/HFD versus HFD/CD *P = 0.0166; P32-35 females CD/CD versus HFD/HFD *P = 0.0189, CD/CD versus HFD/CD *P = 0.0127). Abbreviations: P, postnatal day.

To examine the contribution of maternal HFD during gestation and/or lactation on subsequent offspring, female C57BL/6 J mice were placed on either a HFD (60% calories from fat) or control diet CD (10% calories from fat). Diet was initiated six weeks prior to breeding and continued throughout gestation. The average days between initial mating and pup birth was similar for both CD and HFD-fed dams (mean ± SEM 24.93 ± 1.3 CD versus 25.35 ± 1.6; *P* = 0.8439, two-tailed *t*-test). At birth, dams were either maintained on the same diet or were switched to the opposing diet, generating four groups presented as gestation diet/lactation diet: CD/CD, HFD/HFD, CD/HFD and HFD/CD (Figure 1A). At P21, pups were weaned and placed on standard chow to limit the impact of HFD to the stages associated with maternal care. Dams fed HFD weighed significantly more than CD dams beginning as early as one week after diet initiation and lasting through the last point measured during gestation (Figure 1B). HFD/HFD male and female offspring were significantly heavier at weaning relative to all other conditions within their respective sex (Figure 1C). By P32-35, HFD/HFD male offspring remained significantly heavier than CD/CD and CD/HFD pups but HFD/HFD female offspring were only significantly heavier than CD/CD controls (Figure 1D). Interestingly, at P32-35 both male and female offspring from dams fed HFD during gestation were significantly heavier than CD/CD controls regardless of lactation diet. Examination of overall litter size versus female or male weight revealed no significant correlation between these two factors (Additional file 2: Figure S1). These data demonstrate that maternal HFD significantly impacts maternal and offspring weight during the late juvenile stages.

### Maternal high fat diet during gestation results in increased anxiety in female offspring and hyperactivity in male offspring

**Figure 2:**
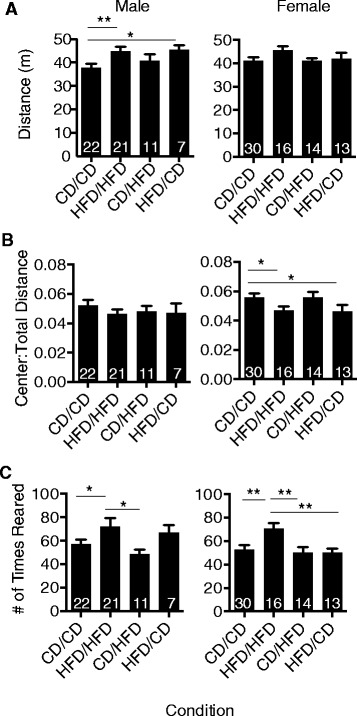
**Maternal obesity increases anxiety in female offspring with partial amelioration by diet intervention.** Male and female offspring from dams fed control diet (CD) or high fat diet (HFD) throughout gestation and lactation or that underwent diet switch at lactation were examined at P32-35 in Open Field Assay (OFA). **(A)** Mean ± SEM for distance traveled (one-way ANOVA with *post-hoc* Fisher’s LSD analysis, male CD/CD versus HFD/HFD **P = 0.0054, CD/CD versus HFD/CD *P = 0.0339). No significant differences were found in female offspring for total distance. **(B)** Center:total distance ratios for male and female offspring shown as mean ± SEM (one-way ANOVA with *post-hoc* Fisher’s LSD analysis, female CD/CD versus HFD/HFD *P = 0.0415, CD/CD versus HFD/CD *P = 0.0362). **(C)** Number of times reared during OFA shown as mean ± SEM number for male and female offspring (one-way ANOVA with *post-hoc* Fisher’s LSD analysis, male CD/CD versus HFD/HFD *P = 0.0386, HFD/HFD versus CD/HFD *P = 0.0134; female CD/CD versus HFD/HFD **P = 0.0025, HFD/HFD versus CD/HFD **P = 0.003, HFD/HFD versus HFD/CD **P = 0.005). N = 7 to 22 or N = 13 to 30 per condition for males and females, respectively, as indicated in the graph. Abbreviations: P, postnatal day.

Maternal HFD maintained throughout gestation and lactation has been associated with increased anxiety in subsequent offspring [14,20,21]. To determine if the gestation and/or lactation periods of maternal diet contributed to increased anxiety, offspring from CD/CD, HFD/HFD, CD/HFD and HFD/CD dams were analyzed in the open field assay (OFA) at P32-35. Maternal diet resulted in a significant increase in distance traveled for male HFD/HFD or HFD/CD versus male CD/CD offspring (Figure 2A). A trend for increased activity in female HFD/HFD offspring was also observed. Female offspring from HFD/HFD and HFD/CD dams relative to CD/CD controls demonstrated a significant decrease in the center:total distance ratio, indicative of increased anxiety, suggesting that the gestational period was critical for altering this behavior (Figure 2B). Increased rearing was also observed within both male and female offspring from HFD/HFD dams (Figure 2C), a sign of increased motility and anxiety. Notably, in female offspring, a striking reduction in rearing behavior was observed upon diet switch at lactation (HFD/HFD versus CD/CD, HFD/HFD versus CD/HFD, HFD/HFD versus HFD/CD), suggesting a partial amelioration of some anxiety measures with maternal dietary intervention (Figure 2C).

### Maternal diet intervention during lactation alleviates social defects in female offspring

In models of maternal immune activation that induce inflammation, ASD-like behaviors such as decreased sociability have been observed [32,46]. As obesity represents a state of low-grade inflammation, we next determined whether maternal obesity impacted sociability and if dietary intervention during the lactation period of maternal diet could alleviate any anomalies in this behavior.

**Figure 3:**
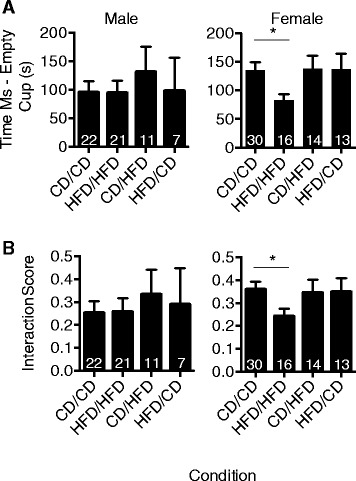
**Maternal high fat diet (HFD) intervention at lactation prevents social abnormalities induced in female offspring by prenatal/postnatal maternal HFD.** Offspring from dams fed HFD or control diet (CD) during gestation and or lactation were examined at approximately P38-42 in the 3-chamber social assay for sociability. **(A)** Mean ± SEM time spent in mouse cup area − time spent in empty cup area for male and females as a measure of social interest (one-way ANOVA with *post-hoc* Fisher’s LSD analysis, *P = 0.029). **(B)** Interaction scores for sociability expressed as mean ± SEM (one-way ANOVA with *post-hoc* Fisher’s LSD analysis, *P = 0.0423). N = 7 to 22 or N = 13 to 30 per condition for males and females, respectively, as indicated in the graph. Abbreviations: P, postnatal day.

At 5.5 to 6 weeks of age, offspring from each group were tested for social interactions using the 3-chamber social test. Alterations in sociability were observed in female offspring, but not male offspring, with HFD/HFD offspring showing a significant reduction in interaction time with cups containing probe mice relative to the empty cups in the CD/CD group (Figure 3A). When normalized for total time spent in both regions, represented as an interaction score, the same significant decrease was observed in HFD/HFD female offspring compared to CD/CD female offspring (Figure 3B). Importantly, HFD/CD dams that received dietary intervention at the lactation stage yielded offspring that were similar in sociability compared to CD/CD dam offspring (Figure 3B). Together, these data indicate that maternal diet impacts offspring sociability and dietary intervention during lactation is sufficient to ameliorate this ASD-like phenotype.

### Decreased microglial activation in female offspring from HFD to CD diet switched dams

**Figure 4:**
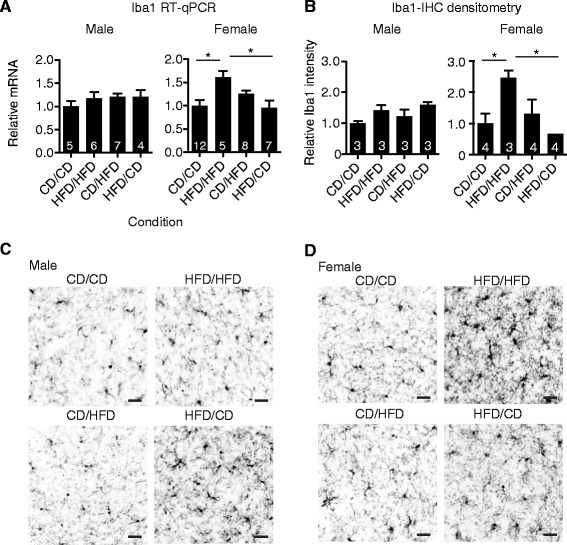
**Increased microglial reactivity induced by maternal obesity is ameliorated by dietary switch during lactation. (A)** Brain microglial *Iba1* mRNA levels from male or female offspring of CD/CD, HFD/HFD, CD/HFD and HFD/CD dams were examined at six weeks of age by quantitative RT-PCR with values depicted as mean ± SEM. N = 4 to 7 for males, N = 5 to 12 per condition for females as indicated in the graph (one-way ANOVA with *post-hoc* Tukey analysis, CD/CD versus HFD/HFD *P = 0.019, HFD/HFD versus HFD/CD *P = 0.0187. **(B-D)** Immunohistochemistry for microglial Iba1 reactivity was conducted on 50-μm amygdala brain sections. **(B)** Iba1 protein was analyzed by densitometry in males and females (N = 3 to 4 per condition as indicated in bars; one-way ANOVA with *post-hoc* Tukey analysis, female CD/CD versus HFD/HFD *P = 0.0394, HFD/HFD versus HFD/CD *P = 0.0119). Representative Iba1 staining from **(C)** male and **(D)** female offspring. Scale bars = 25 μm. Abbreviations: CD, control diet; HFD, high fat diet; P, postnatal day.

Microglia are brain resident immune cells that can modulate the cytokine *milieu* and impact neuronal function [47-50]. To determine if dietary intervention can reduce maternal HFD-induced microglial activation in offspring, gene expression of *Iba1* within brains of male or female offspring from CD/CD, HFD/HFD, CD/HFD and HFD/CD dams was examined by RT-qPCR. Maternal dietary conditions had no impact on *Iba1* gene expression within male offspring. In contrast, within female offspring, a significant increase in *Iba1* transcript was observed in HFD/HFD relative to CD/CD female offspring controls (Figure 4A). Lowering maternal dietary fat content during lactation resulted in a significant decrease in *Iba1* transcripts in the brain (HFD/HFD versus HFD/CD). Iba1 immunohistology in the amygdala, a region associated with anxiety and social behaviors, was similar over different maternal dietary conditions in male offspring (Figure 4B,C). However, Iba1 staining revealed that microglia from HFD/HFD female offspring appeared more reactive relative to CD/CD, CD/HFD and HFD/CD female offspring (Figure 4D). Densitometric analysis also showed significantly more Iba1 staining intensity in HFD/HFD female offspring compared to CD/CD controls as well as HFD/CD female offspring (Figure 4B). Therefore, maternal HFD likely impacts the microglial population in female offspring and dietary intervention during the lactation period reduces reactivity.

### Maternal HFD induces a pro-inflammatory *milieu* in the brain that is altered by dietary intervention

**Figure 5:**
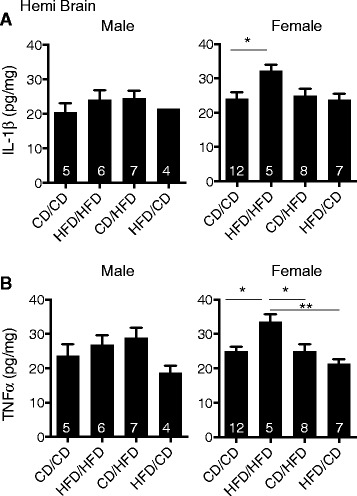
**Maternal dietary intervention reduces pro-inflammatory cytokines in the central nervous system (CNS) of offspring from maternally obese dams. (A)** IL-1β and **(B)** TNFα protein levels in brains from male or female offspring were examined at six weeks of age by ELISA. Values are depicted as mean ± SEM with N = 4 to 7 for males and N = 5 to 12 per condition for females as indicated in the graph. One-way ANOVA with *post-hoc* Tukey analysis, female IL-1β CD/CD versus HFD/HFD *P = 0.0385; female TNFα CD/CD versus HFD/HFD *P = 0.0106, HFD/HFD versus CD/HFD *P = 0.0182, HFD/HFD versus HFD/CD **P = 0.0008. No significant differences were found in male offspring. Abbreviations: CD, control diet; HFD, high fat diet; P, postnatal day.

To examine whether the pro-inflammatory cytokine *milieu* corresponded with altered sociability and microglial reactivity patterns, brain lysates of offspring from each group were examined for IL-1β and TNFα levels. No significant differences in either IL-1β or TNFα were detected within male offspring from the different maternal dietary conditions (Figure 5A,B). As was observed with social readouts, female HFD/HFD offspring displayed an elevated level of inflammation in brain tissue (Figure 5A,B). Female HFD/HFD offspring had significantly higher IL-1β and TNFα levels compared to female CD/CD controls. Again, dietary intervention at the point of lactation (that is HFD/CD) resulted in offspring with similar cytokine levels to CD/CD dam offspring and, in the case of TNFα, was significantly lower than HFD/HFD dam offspring. Together, these data indicate that dietary intervention is able to alleviate the impact of maternal HFD on offspring brain inflammation.

## Discussion

Obesity is rising to staggering proportions in the US [1] and impacts several aspects of human health. While obesity is a comorbidity for several diseases, including diabetes, cancer, and cardiovascular disease [51], studies now also show associations between obesity and altered cognition and behavior [52]. Additionally, maternal HFD and obesity can have significant ramifications in subsequent offspring [13,14,20,21], indicating the importance of pre- and perinatal maternal obesity. It is therefore critical to understand how maternal obesity influences offspring and whether efficacious intervention strategies exist. Here we show that maternal HFD can significantly impact offspring CNS inflammation, hyperactivity, anxiety, and sociability. Critically, we find that maternal dietary intervention during lactation is sufficient to offset many of the maladaptive responses observed in subsequent offspring.

Pre-pregnancy adiposity is also associated with development of ADHD symptoms in children [22-24]. In accordance with a previous study [25], we demonstrated that maternal HFD contributes to hyperactivity in subsequent male HFD/HFD and HFD/CD offspring relative to CD/CD controls (Figure 2A). Female HFD/HFD offspring showed a similar trend toward increased activity. Maternal HFD feeding during gestation alone was sufficient to induce this response, indicating that this is a sensitive developmental period for hyperactivity phenotypes. Interestingly, increased levels of pro-inflammatory cytokines were not observed across groups in male offspring (Figure 5A,B), suggesting that the hyperactivity phenotype may be associated with a separate parameter. Recently, hyperactivity and impulsivity symptoms in ADHD children have been associated with altered dopaminergic and serotonergic pathways [53] and, in primates, maternal HFD has also been shown to alter offspring serotonergic pathways [19]. This suggests that gestational maternal obesity may alter hyperactivity through long-lasting modifications of the serotonergic pathway.

In other behavioral parameters, we found that maternal HFD during gestation and lactation resulted in significant alterations only within female offspring. In humans, maternal obesity is associated with increased risk of autism (odds ratio = 1.67) [30]. Accordingly, we show that female offspring from HFD/HFD dams had reduced sociability compared to controls (Figure 3A,B). Although ASD-like behaviors have been shown in prenatal infection models [31,32,46], our data demonstrate social deficits can occur as a consequence of maternal obesity alone in a rodent model. The interaction score used to assess social interaction normalizes for the total time in both the mouse and empty cup region to more accurately reflect measures of social anxiety rather than a failure to exhibit exploratory behavior. The factors by which maternal obesity decreases offspring sociability are unknown; however, obesity-induced low-grade chronic inflammation likely plays a role in altered offspring behavior. Notably, in humans, obese mothers have increased IL-6 in plasma and breast milk and increased IL-6 gene expression in placental macrophages [54-56]. In rodents, increased fetal IL-6 levels from high fat/sugar-fed dams are also observed [57]. Prenatal exposure to IL-6, either through maternal immune activation (MIA) or cytokine injection, is sufficient to cause aberrant ASD-like behaviors and altered brain transcriptomes in offspring [58,59]. Therefore, increased maternal IL-6 likely plays a role in decreased sociability in offspring from obese dams. Although unexplored in our study, maternal obesity also impacts many other aspects of offspring brain homeostasis, such as serotonin pathways, lipid peroxidation, and corticosteroid receptor expression [12,13,19,21] that may also contribute to aberrations in offspring behavior.

In human children and adults, obesity is associated with increased anxiety and associations are sometimes stronger in females [16,57]. Examination of non-human primate offspring from maternally obese mothers also revealed a female bias for elevated anxiety [19]. Likewise, our data demonstrate an increase in anxiety and decreased sociability only within female offspring from HFD/HFD dams (Figure 2B,C). In other rodent models, maternal HFD increased anxiety in both male and female offspring, measured by decreased time spent in the center region of the OFA or the percentage of time in the open arms of elevated plus maze [14,20,21]. Although the reason for discrepancies in affected sex is unclear, in some cases [14,21] this may be a reflection of differences in rat versus mouse models. Previous work has demonstrated developmental sex discrepancies in rat brain microglial colonization [60]. Increased microglial numbers in early postnatal male rat brains may increase susceptibility to early life insults. Currently, it is unknown if mice have similar sex differences in microglial colonization; however, at P3, increased *Il1b*, *Tnfa*, and *Il6* gene expression has been shown in female brains [61], suggesting that during early mouse development, female brains have a more pro-inflammatory *milieu* compared to males. This may be important since elevated CNS pro-inflammatory cytokines, including IL-1β and/or TNFα, have been associated with increased anxiety, decreased cognition, and altered social behaviors [35-38,62]. Our data demonstrated that elevated brain inflammation corresponded to behavioral manifestations. Significant increases in brain levels of both IL-1β and TNFα were observed only in female offspring from HFD/HFD dams (Figure 5) that were also the only group to display increased anxiety and decreased sociability (Figures 2 and 3). Notably, within male offspring no alterations were observed between groups in regard to neuroinflammation (Figures 4 and 5) or anxiety and sociability parameters (Figures 2 and 3), further supporting the idea of a link between pro-inflammatory cytokines and behavioral deficits in female offspring. Additionally, increased hippocampal IL-1β has been observed in offspring of both sexes with altered behavior [14]. Therefore, regardless of differences in the impacted sex between studies, maternal obesity results in increased offspring CNS inflammation that is associated with behavioral abnormalities.

Microglia are integral resident CNS immune cells that are critical for both homeostatic and pathological states of the CNS [47-50]. Microglia continually survey the brain parenchyma and, upon activation, are capable of rapidly redirecting processes, phagocytosis, and augmenting inflammation via production of reactive oxygen species and pro-inflammatory cytokines including IL-1β and TNFα, ([63], 2005; [47,64]). Our data revealed increased brain gene expression and amygdala tissue qualitative and quantitative protein expression of the microglial marker, ionized calcium binding adaptor molecule 1 (Iba1) (Figure 4A,B,D) in female offspring from HFD/HFD dams. This is suggestive of increased microglial activation. However, it is interesting to note that, unlike what was observed within female offspring, altered *Iba1* mRNA and protein expression was not observed in male offspring across conditions (Figure 4A,B) that also showed no signs of neuroinflammation (Figure 5A,B). Together with previously reported increased offspring hippocampal Iba1 expression after maternal saturated fat feeding [14], these data suggest that microglial reactivity in multiple brain regions are altered by prenatal/perinatal maternal dietary conditions. Because microglia can self-renew for CNS maintenance [47,65], early insults may have a long-lived impact. Notably, in perinatal bacterial infection models, microglial Iba1 immunoreactivity was associated with increased anxiety and elevated brain IL-1β levels that corresponded with decreased memory after lipopolysaccharide (LPS) challenge [35,43,66]. Isolated CD11b^+^ microglia from LPS injected mice, previously exposed to perinatal infection, demonstrated increased IL-1β secretion, supporting the idea that early exposure to inflammation has long- lasting consequences on microglial reactivity [43]. Although microglia-specific IL-1β and TNFα levels were not specifically examined, maternal obesity likely acts as an early life insult that alters microglial reactivity, allowing them to contribute to elevated offspring brain inflammation.

One of our most important findings is that dietary intervention during lactation alleviates the effects of gestational HFD. Offspring from HFD dams switched to CD during lactation no longer exhibit aberrations in rearing and social behavior (Figures 2 and 3). Additionally, maternal dietary intervention ameliorated microglia activation and brain pro-inflammatory cytokines observed in offspring of HFD/HFD dams (Figures 4 and 5). An important *caveat* to note in our study is that rodent and human brain development occurs at different stages with the peaks of gliogenesis and brain growth spurts occurring postnatally in rodents versus prenatally in humans [67]. Therefore, future studies examining diet regiments that are altered later during lactation in rodents, after the majority of glial development is completed, may be more translatable to the human condition. It is likely that early prenatal dietary interventions will have the best efficacy in humans. While the mechanism by which dietary intervention exerts these effects in rodents is unknown, intervention may modulate epigenetic changes caused by maternal diet or impact offspring exposure to maternal pro-inflammatory cytokines or maternal microbiomes. Alterations in methyl-CpG-binding protein 2 (MeCP2) results in ASD-like behaviors [68-70]. Since MeCP2 binds to methylated DNA, epigenetic changes may alter MeCP2 binding and function. Therefore, it is interesting to note that maternal HFD has been shown to alter methylation in the CNS [71], although it is unknown if it specifically impacts MeCP2 targets related to ASD-like behaviors. Obesity and HFD significantly shift intestinal microbiota [72-76] and increased BMI is associated with altered, less diverse microbiomes and elevated IL-6 in breast milk [55]. Microbial colonization is thought to occur after birth, making the postnatal period critical for shaping the subsequent microbiome [77]. Interestingly, parental diet has been shown to modulate the microbiome of subsequent offspring [78] and emerging evidence has demonstrated that the microbiome composition can significantly impact immunity, inflammation, and obesity [78-81]. Both chronic stress and autism have been associated with altered microbiomes, and postnatal colonization is implicated in modulation of the hypothalamic-pituitary-adrenal axis suggesting links between brain function and intestinal bacteria [82-84]. Whether dietary intervention is able to shift offspring microbiome to elicit behavioral effects will require further experiments.

In summary, these data demonstrate that maternal obesity results in altered anxiety, hyperactivity, and for the first time, sociability in offspring. Neuroinflammation, reflected by elevated IL-1β, TNFα, and microglial Iba1 staining and transcripts in affected female offspring, may be a mechanism for the enduring behavioral alterations as a result of pre- and postnatal exposure to maternal HFD. Importantly, our data highlight dietary intervention as a potential therapeutic strategy to offset the deleterious effects of maternal obesity on offspring behavior and CNS inflammation.

## Additional files

Additional file 1: Table S1. Comparison of control and high fat diet formulations. Additional file 2: Figure S1. Litter size does not impact offspring weight. Offspring weight was compared to the litter size in A, male and female offspring at P21 and B, male and female offspring at P32-35. Linear regression analysis revealed no significant correlations in all groups, N = 73 and N = 61 for males and females, respectively, at both timepoints.

## Abbreviations

AHDH: Attention deficit hyperactivity disorder
ANOVA: Analysis of variance
ASD: Autism spectrum disorder
BMI: Body mass index
CNS: Central nervous system
DTT: Dithiothreitol
ELISA: Enzyme-linked immunosorbent assay
HFD: High fat diet
Iba1: Ionized calcium binding adaptor molecule 1
IL: Interleukin
LPS: Lipopolysaccharide
MeCP2: Methyl-CpG-binding protein 2
MIA: Maternal immune activation
NBF: Normal buffered formalin
OFA: Open field assay
P: Postnatal day
PBS: Phosphate buffered saline
PBS-X: phosphate buffered saline with triton-X
RT: Room temperature
RT-qPCR: real time quantitative polymerase chain reaction
TBSE: Tris buffered saline with EDTA
TNF: Tumor necrosis factor
