# Supplementary material for: Dietary intervention rescues maternal obesity induced behavior deficits and neuroinflammation in offspring
Source: J Neuroinflammation. 2014 Sep 12;11:156. doi: 10.1186/s12974-014-0156-9 (PMC4172780; doi:10.1186/s12974-014-0156-9)
Supplement: Additional file 1: Table S1. — Comparison of control and high fat diet formulations. [file 12974_2014_156_MOESM1_ESM.pdf]

Supplemental Table 1. Comparison of control and high fat diet formulations.

		control diet #D12450B		high fat diet #D12492	
		gm%	kcal%	gm%	kcal%
protein		19.2	20	26.2	20
carbohydrate		67.3	70	26.3	20
fat		4.3	10	34.9	60
	Total kcal/gm	3.85	100	5.24	100
ingredients		gm	kcal	gm	kcal
casein, 30 Mesh		200	800	200	800
L-Cystine		3	12	3	12
Corn starch		315	1260	0	0
Maltodextrin 10		35	140	125	500
Sucrose		350	1400	68.8	275.2
Cellulose, BW200		50	0	50	0
Soybean oil		25	225	25	225
Lard		20	180	245	2205
Mineral mix S10026		10	0	10	0
DiCalcium phosphate		13	0	13	0
Calcium carbonate		5.5	0	5.5	0
Potassium citrate		16.5	0	16.5	0
Vitamin mix V10001		10	40	10	40
Choline bitartrate		2	0	2	0
FD&C Yellow Dye #5		0.05	0	0.05	0
**Total**		1055.5	4057	773.85	4057
