# Supplementary figures and images for: Dietary intervention rescues maternal obesity induced behavior deficits and neuroinflammation in offspring
Source: J Neuroinflammation. 2014 Sep 12;11:156. doi: 10.1186/s12974-014-0156-9 (PMC4172780; doi:10.1186/s12974-014-0156-9)

**A** P21

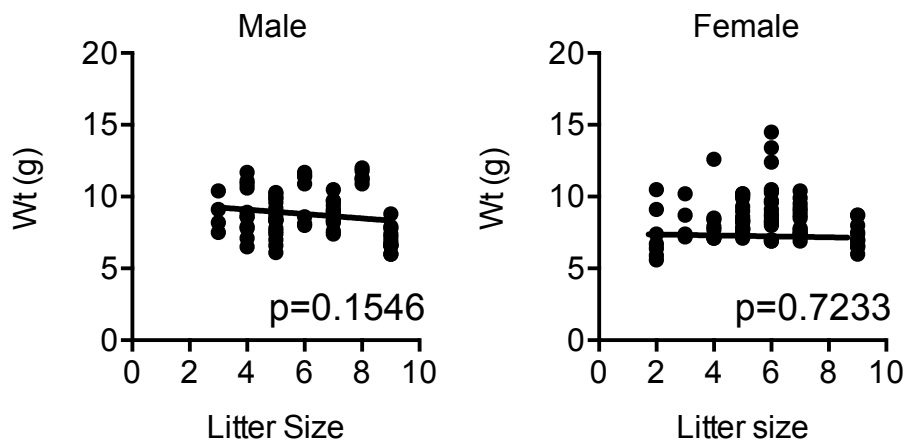

**B** P32-35

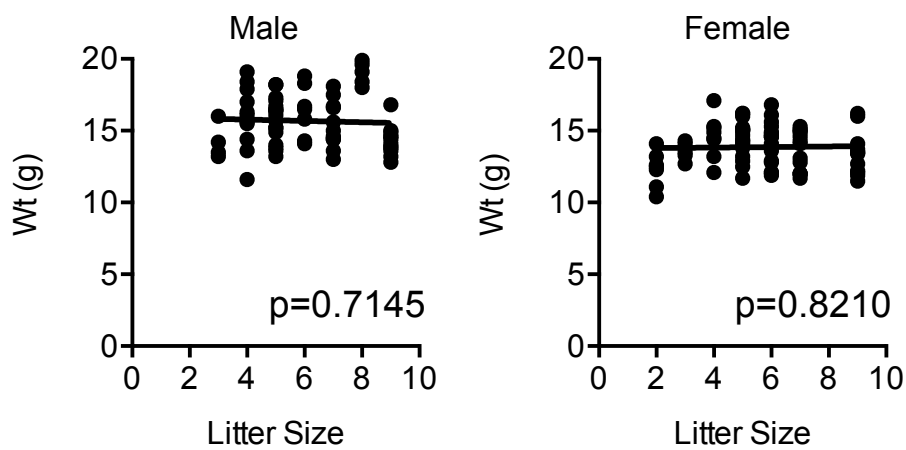

Supplement: Additional file 2: Figure S1. — Litter size does not impact offspring weight. Offspring weight was compared to the litter size in A, male and female offspring at P21 and B, male and female offspring at P32-35. Linear regression analysis revealed no significant correlations in all groups, N = 73 and N = 61 for males and females, respectively, at both timepoints. [file 12974_2014_156_MOESM2_ESM.pdf]
